# Upstream Distal Regulatory Elements Contact the *Lmo2* Promoter in Mouse Erythroid Cells

**DOI:** 10.1371/journal.pone.0052880

**Published:** 2012-12-21

**Authors:** Anandi Bhattacharya, Chih-Yu Chen, Sara Ho, Jennifer A. Mitchell

**Affiliations:** Department of Cell and Systems Biology, University of Toronto, Toronto, Ontario, Canada; Southern Illinois University School of Medicine, United States of America

## Abstract

The Lim domain only 2 (*Lmo2*) gene encodes a transcriptional cofactor critical for the development of hematopoietic stem cells. Several distal regulatory elements have been identified upstream of the *Lmo2* gene in the human and mouse genomes that are capable of enhancing reporter gene expression in erythroid cells and may be responsible for the high level transcription of *Lmo2* in the erythroid lineage. In this study we investigate how these elements regulate transcription of *Lmo2* and whether or not they function cooperatively in the endogenous context. **C**hromosome conformation capture (3C) experiments show that chromatin-chromatin interactions exist between upstream regulatory elements and the *Lmo2* promoter in erythroid cells but that these interactions are absent from kidney where *Lmo2* is transcribed at twelve fold lower levels. Specifically, long range chromatin-chromatin interactions occur between the *Lmo2* proximal promoter and two broad regions, 3–31 and 66–105 kb upstream of *Lmo2*, which we term the proximal and distal control regions for *Lmo2* (pCR and dCR respectively). Each of these regions is bound by several transcription factors suggesting that multiple regulatory elements cooperate in regulating high level transcription of *Lmo2* in erythroid cells. Binding of CTCF and cohesin which support chromatin loops at other loci were also found within the dCR and at the *Lmo2* proximal promoter. Intergenic transcription occurs throughout the dCR in erythroid cells but not in kidney suggesting a role for these intergenic transcripts in regulating *Lmo2*, similar to the broad domain of intergenic transcription observed at the human β-globin locus control region. Our data supports a model in which the dCR functions through a chromatin looping mechanism to contact and enhance *Lmo2* transcription specifically in erythroid cells. Furthermore, these chromatin loops are supported by the cohesin complex recruited to both CTCF and transcription factor bound regions.

## Introduction

Lim domain only 2 (LMO2) is a critical transcriptional regulator of hematopoiesis. Gene targeting experiments conducted to introduce null mutations in the mouse *Lmo2* gene, have shown that *Lmo2* is necessary for embryonic yolk sac erythropoiesis [1]. During differentiation of hematopoietic progenitor cells, *Lmo2* expression is maintained in erythroid cells but down regulated in the T-cell lineage [1], [2], [3], [4]. Aberrant expression of *Lmo2* results in the development of T-cell related diseases; indeed *Lmo2* is located at a recurrent site of T-cell acute lymphoblastic leukemia (T-ALL) specific translocation [2], [4], [5], [6], [7], [8]. In addition, patients undergoing gene therapy for X-linked severe combined immunodeficiency developed clonal T-cell proliferation as a result of aberrant transcriptional activation of *Lmo2* when the gene therapy vector integrated near *Lmo2* [9].

Previous studies have shown that in erythroid cells LMO2 is usually present as part of a complex with the transcriptional regulators, TAL1, E47, LDB1, and GATA1 [10], [11], [12]. This protein complex binds DNA by recognizing a bipartite DNA sequence comprising of an E box and a GATA site [10], [12]. These LMO2 containing oligomeric complexes along with other factors in hematopoietic cells have been found on the regulatory regions of various other genes including, β-globin (*Hbb*), α-globin (*Hba*), retinaldehyde dehydrogenase 2, *c-kit* and erythroid Kruppel-like factor (*Eklf*) [13], [14], [15], [16], [17], [18].

Transcription is regulated not only by the sequences immediately upstream of gene transcription start sites (TSS) but in many cases by distal regulatory elements (DRE) which can be located up or downstream of the genes they regulate. In fact genome-wide chromatin immunoprecipitation sequencing (ChIP-Seq) analysis for several transcription factors has revealed that a significant proportion (40–60%) of transcription factor bound regions are located in the intergenic regions of the genome ≥10 kb from a gene TSS [19], [20], [21]. In general DRE with enhancer activity are associated with increased sensitivity to DNaseI, co-binding of multiple transcription factors, binding of the histone acetyl transferase p300, increased histone H3 monomethylation of lysine 4 (H3K4me1), increased histone H3 acetylation of lysine 27 (H3K27ac) and recruitment of the mediator and cohesin complexes [21], [22], [23], [24], [25], [26], [27], [28], [29]. There are many examples of regulatory elements located several kilobases from their target genes, including the well studied regulatory elements of the *Hbb* locus control region (LCR) [30]. The LCR consists of a series of transcription factor bound DNaseI hypersensitive sites 50 kb upstream of the *Hbb-b1* gene [31], [32], [33]. Chromosome conformation capture (3C) in adult erythroid cells has revealed that the *Hbb* LCR is in close proximity to the active *Hbb* genes *(Hbb-b1* and *Hbb-b2*) whereas the intervening 50 kb of DNA sequence containing the embryonic erythroid cell expressed genes is looped out [34], [35], [36]. The 3C technique has since been used to detect chromatin-chromatin interactions between DRE and several genes including: *Hba*, *Shh*, T_H_2, *HoxB1* and olfactory receptor genes [37], [38], [39], [40], [41].

The chromatin-chromatin looping interactions that regulate cell-type specific gene expression are also present in a cell-type specific manner whereas many of the proteins present at sites of looping interactions are ubiquitously expressed. For example, CTCF participates in intra- and inter-chromosomal looping at individual gene loci including *Hbb*, *Igf2/H19* and *HoxA*, however, CTCF bound regions are generally bound by CTCF in all cell types [42], [43], [44], [45]. Genome-wide studies have shown that CTCF and cohesin, a protein complex that mediates sister chromatid cohesion, localise to the same regions of the genome [46]. Furthermore, at the imprinted *IGF2-H19* locus and at the developmentally regulated IFNG locus both cohesin and CTCF are required for maintaining higher-order chromatin conformation [47], [48]. Whereas the CTCF bound regions of the genome show limited differences between cell types, CTCF/cohesin bound regions do form tissue specific chromatin loops [42], [49]. In addition, the presence of cohesin correlates with the number of bound transcription factors at *cis*-regulatory elements not occupied by CTCF [50]. Members of the cohesin complex also interact with mediator, a complex recruited by transcription factors which acts as a bridge to the RNA polymerase II preinitiation complex [24], [51]. Both mediator and cohesin proteins are bound at enhancers that form ES-cell specific chromatin loops with a nearby gene promoter [24]. In summary the cohesin complex appears to have a critical role in mediating chromatin loop formation at both CTCF bound regions of the genome and transcription factor bound distal regulatory elements required for tissue-specific gene transcription [48], [52].

Transcriptional regulation of *Lmo2* in erythroid cells appears to involve multiple distal regulatory elements. Three alternate upstream promoters have been identified as well as multiple conserved DRE located up to 100 kb upstream of the *Lmo2* gene [53], [54], [55], [56]. DRE located 90, 75, 64, 25 and 12, kb upstream of *Lmo2* and 1 kb downstream are capable of enhancing reporter gene expression in erythroid cells and in transgenic mice suggesting that strong expression of *Lmo2* in hematopoietic cells requires the combined action of upstream DRE and sequences close to the *Lmo2* proximal promoter [54]. In transgenic mice optimal expression in fetal liver and circulating erythrocytes was obtained using a multi-enhancer construct containing the 75, 70, 25, 12 and +1 DRE in conjunction with an extended proximal promoter region (pPex). Here we investigate long-range regulation of *Lmo2* transcription in the context of its endogenous genomic location using the 3C technique. Our results show that multiple upstream DRE interact with the *Lmo2* proximal promoter whereas intervening regions are looped away from the proximal promoter. DRE upstream of *Lmo2* are bound by multiple transcription factors, p300, and associated with intergenic transcription when *Lmo2* is transcribed at high levels in erythroid cells. Interaction between the DRE and the *Lmo2* promoter was identified in erythroid cells but not in kidney cells suggesting a link between the looping conformation of the locus and transcriptional regulation of *Lmo2*. Furthermore, a CTCF and cohesin occupied region upstream of the most distal enhancer (90 DRE) also contacts the *Lmo2* proximal promoter region, potentially insulating the neighboring Cell cycle associated protein 1 (*Caprin1*) gene from interaction with the DRE that enhance *Lmo2* transcription.

## Results

### Distal regulatory elements upstream of Lmo2 overlap transcription factor bound regions

**Figure 1:**
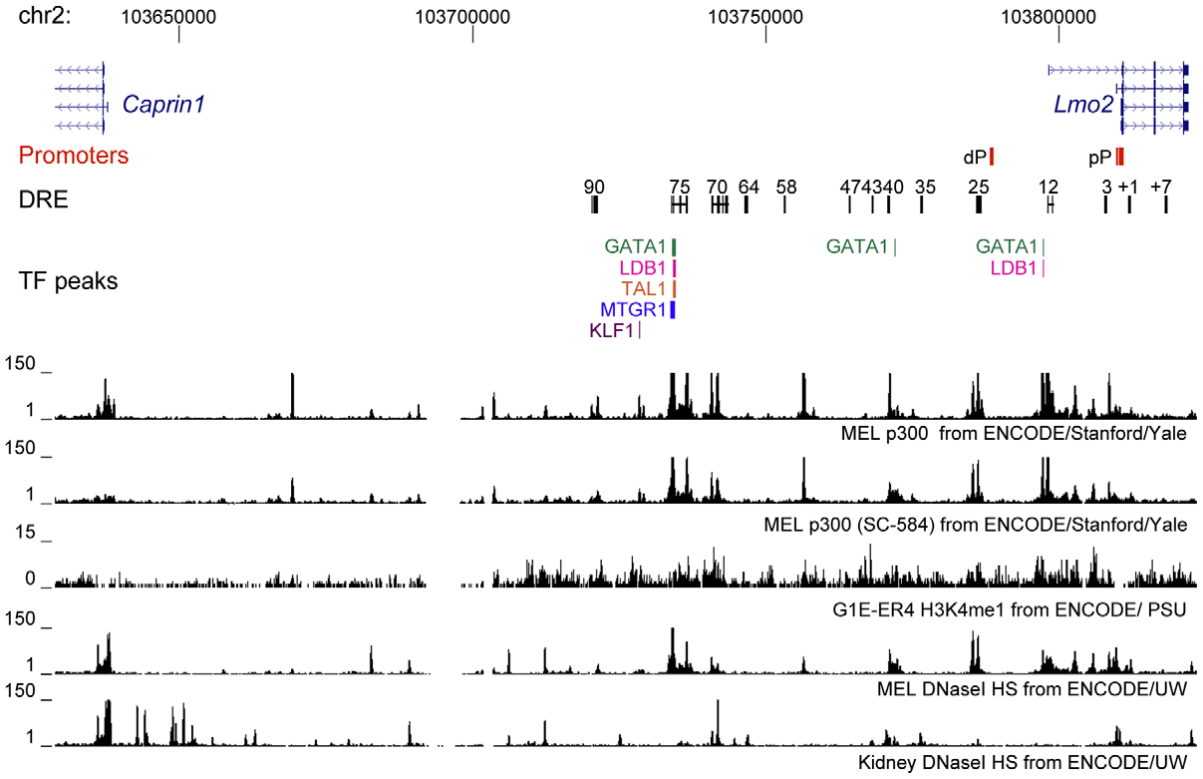
Distal regulatory elements upstream of *Lmo2* overlap transcription factor bound regions in erythroid cells. The mouse *Lmo2-Caprin1* region on chromosome 2 is depicted with chromosome coordinates shown at the top. Two *Lmo2* promoters are indicated by red boxes. Distal regulatory element (DRE) homology regions are indicated by black boxes joined by a line to delineate the human enhancer construct used in the generation of transgenic mice. Mouse ENCODE ChIP-Seq data for p300 and DNaseI hypersensitivity are shown below the DRE track. Coloured boxes represent peaks identified from transcription factor ChIP-Seq data for erythroid (MEL and GIE-ER4) cells. Overlapping transcription factor peaks were identified at the 75 and 12 DRE. These regions were also occupied by p300 and showed increased sensitivity to DNaseI. The entire locus was marked with histone H3 lysine 4 monomethylation (H3K4me1). Proximal promoter (pP), distal promoter (dP), murine erythroleukemia cells (MEL), Transcription factors (TF).

Multiple DRE upstream of *Lmo2* have been identified in the human genome and confirmed to have enhancer activity in transgenic mice [54]. We mapped the proximal and distal promoters and enhancer sequences identified by Landry et al. 2009 to the mouse genome and located these sequences within a region 90 kb upstream and 7 kb downstream of *Lmo2* (Figure 1, Tables S1 and S2). The intermediate promoter TSS for *Lmo2*, identified in the human genome and found to confer expression in human T-ALL cell lines that express *LMO2*, is marked by the top transcript in the gene annotation track [53]. Several transcription factors (LMO2, TAL1, GATA2, FLI1, and SFPI1) have been found associated with these DRE using a ChIP-chip approach [54]. We retrieved available ChIP-Seq data for erythroid cells (KLF1, MTGR1, GATA1, TAL1, LDB1, Table S3) to more finely map the transcription factor-bound regions within each DRE (Figure 1) [57], [58], [59]. Our analysis revealed several transcription factors (MTGR1, GATA1, TAL1, LDB1) bound at the 75 DRE (Figure 1). Transcription factors were also bound near the 40 (GATA1) and 12 (GATA1, LDB1) DRE. In addition to transcription factors, DRE with enhancer activity are associated with p300 and increased DNaseI sensitivity [22], [25], [26], [60]. Using available ChIP-Seq data from the mouse ENCODE project (Table S3) [61] we identified p300 and DNaseI hypersensitivity specific to erythroid cells at the 75, 25 and 12 DRE. In contrast H3K4me1, another feature associated with enhancer regions, was found to be generally increased through the 100 kb region encompassing the previously identified DREs near *Lmo2*.

### The 75 distal regulatory element contacts the *Lmo2* proximal promoter

**Figure 2:**
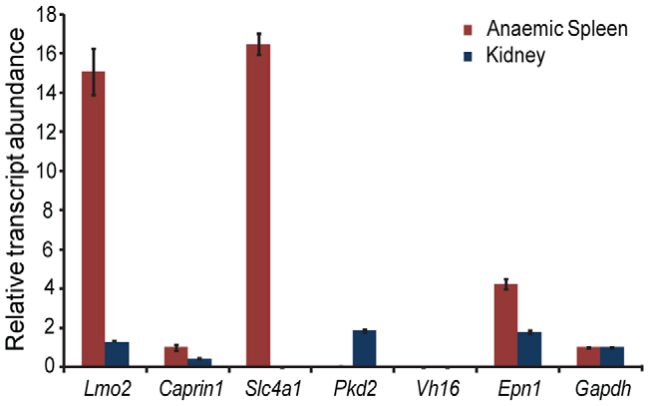
*Lmo2* primary transcripts are abundant in erythroid cells. Primary transcript levels in adult mouse anaemic spleen (red) and kidney (blue) for: *Lmo2* (exon2-intron2), *Caprin1* (exon3-intron2), *Slc4a1* (exon1-intron1), *Pkd2* (intron2-exon3), *Epn1*(exon1-intron1), *Gapdh* (exon1-intron1) and *Vh16 (genic)*. Levels were quantitatively assessed by RT-qPCR and expressed relative to *Gapdh*. *Epn1* is a second ubiquitously expressed reference gene, *Slc4a1* is an erythroid cell specific transcript, *Pkd2* is a kidney specific transcript, *Vh16* is not expressed in either tissue.

To investigate whether or not chromatin loops form in erythroid cells which bring the 75 DRE into proximity with the *Lmo2* promoter we performed 3C in adult erythroid cells isolated from mouse anemic spleens 5 days after the initiation of Phenylhydrazine treatment [62]. On day five the anaemic spleen is composed of >85% globin expressing erythroid cells [63]. For comparison we used kidney as a tissue in which *Lmo2* is not transcribed at robust levels. We confirmed robust transcription of *Lmo2* in isolated anaemic spleen by measuring the levels of the primary transcript by RT-qPCR (Figure 2). By contrast *Lmo2* primary transcript levels were twelve fold lower in kidney. We also examined the primary transcript levels of the cell cycle associated protein *Caprin1* that is located 172 Kb upstream of *Lmo2* with the DREs upstream of *Lmo2* located between the two genes. We found that *Caprin1* is transcribed in both adult erythroid and kidney cells with primary transcript levels 2 fold higher in adult erythroid cells compared to kidney.

**Figure 3:**
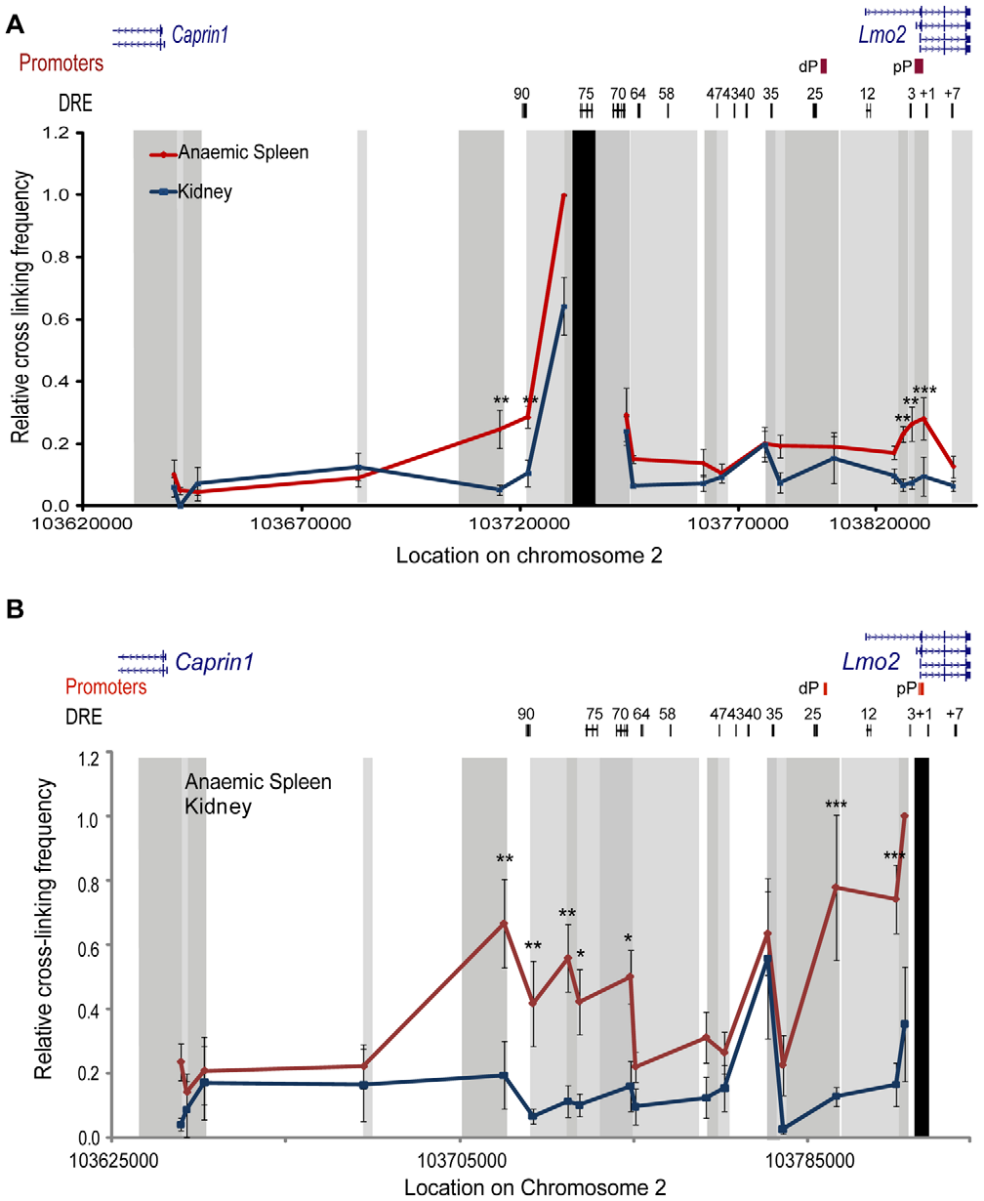
Distal regulatory elements interact with the *Lmo2* proximal promoter in erythroid cells. **A**) Quantitative chromosome conformation capture (3C) was performed to detect chromatin-chromatin interactions between the 75 DRE (distal regulatory element) upstream of *Lmo2* and the rest of the *Lmo2*-*Caprin1* region of mouse chromosome 2. B) Similarly, 3C was performed to detect chromatin-chromatin interactions between the *Lmo2* proximal promoter (pP) and distal regulatory elements (DRE). In both the profile of interactions identified in anaemic spleen (red) and kidney (blue) is displayed. Black box indicates the anchor fragment at the 75 DRE or the *Lmo2* pP and alternating intensities of grey boxes indicate the fragments investigated for interactions. Data points are an average of three to five independent biological replicates. Error bars depict the SEM, * p<0.05, ** p<0.01, and *** p<0.001.

As our analysis of the ChIP-Seq data revealed the highest density of transcription factors bound at the 75 DRE we performed 3C experiments using the *HindIII* fragment containing the 75 DRE as the anchor fragment (Figure 3A, restriction fragment map and primers shown in Figure S1). 3C analysis revealed significantly increased interaction between the 75 DRE and the *Lmo2* proximal promoter fragment in erythroid cells compared to kidney tissues. Of note the *Lmo2* proximal promoter fragment also contains the +1 enhancer element found to cooperate with the 75 DRE for optimal expression in circulating erythrocytes of transgenic mice [54]. We also found increased interaction of the 75 DRE with the two fragments upstream of the *Lmo2* proximal promoter in erythroid cells compared to kidney. This region contains the 3 DRE identified by Landry et al. 2009 which appeared not to have enhancer activity in transgenic analysis. We did not identify interaction of the 75 DRE with the fragment containing the 25 DRE/distal promoter or the 12 DRE/intergenic promoter. Interestingly, the 75 DRE was found to have significantly increased interaction with a fragment overlapping the 90 DRE and a fragment upstream of the 90 DRE in erythroid cells compared to kidney. We found no interaction between the 75 DRE and the *Caprin1* TSS fragment. These results indicate that chromatin-chromatin interactions between 75 DRE and the *Lmo2* proximal promoter and +1 enhancer occur when *Lmo2* is transcribed in erythroid cells but not in kidney where the gene is transcribed at twelve fold lower levels (Figure 2).

### Several upstream distal regulatory elements contact the *Lmo2* promoter

Next we performed a locus-wide 3C using the *Lmo2* proximal promoter as the anchor fragment. We found significantly increased interaction between the *Lmo2* proximal promoter and the fragments containing the 12, 25, 70, 75 and 90 DRE in erythroid cells compared to kidney cells (Figure 3B). These findings indicate that two broad domains interact with the *Lmo2* proximal promoter in a tissue specific manner. The first domain, which we term the proximal control region (pCR), is located close to the *Lmo2* proximal promoter and contains the distal and intermediate promoters as well as the 12 and 25 DRE. The second interaction domain located further upstream of *Lmo2*, which we term the distal control region (dCR), contains the 70, 75 and 90 DRE as well as a fragment upstream of the 90 DRE. These are similar, though not identical, to clusters I (−90 to −64) and II (−40 to +1) identified by Landry et al. 2009 as enriched in histone H3 acetylation in erythroid cells [54]. We also detected a peak in the relative interaction frequency at the 35 DRE; however this interaction was detected in both kidney and erythroid cells (Figure 3B).

Our initial analysis of transcription factors bound in the intergenic region between *Caprin1* and *Lmo2* highlighted the 75 DRE as being bound by multiple transcription factors but did not reveal binding at the 90 and 70 DRE. By contrast our 3C data revealed a broad domain of chromatin-chromatin contacts between the 70, 75 and 90 DRE and the *Lmo2* proximal promoter raising questions about the function of this dCR. We did identify additional p300 association at the 70 DRE suggesting that bound transcription factors are present which recruit p300 to this DRE (Figure 1). To investigate additional transcription factors bound upstream of *Lmo2* we retrieved transcription factor ChIP-Seq data for the HPC7 hematopoietic progenitor cell line: ERG, FLI1, GATA2, GFI1B, LMO2, MEIS1, PU1, TAL1, and RUNX1 [64]. This data revealed multiple transcription factors associated with other DRE in the intergenic region including the 90, 75, 70, 64, 40, 25 and 12 DRE (Figure S2).

### The *Caprin1* TSS does not interact with the identified distal regulatory elements

*Caprin1* is a ubiquitously expressed gene located 172 kb upstream of *Lmo2* and transcribed from the opposite strand. As *Caprin1* is transcribed in erthyoid cells we were interested to investigate whether or not *Caprin1* physically interacts with the DRE located between *Caprin1* and *Lmo2*. Our initial 3C experiments performed with the 75 DRE as the anchor fragment did not show any increase in the relative interaction frequency between the *Caprin1* TSS and the 75 DRE (Figure 3A), however as *Caprin1* could physically interact with other DRE located between *Caprin1* and *Lmo2* we performed 3C experiments using the *HindIII* fragment containing the *Caprin1* TSS as the anchor fragment. Our results did not show any significant peaks in the relative interaction frequency with the *Caprin1* TSS in the entire region between the *Caprin1* and *Lmo2* genes (Figure S3). Furthermore we identified no significant differences between the relative interaction frequency of any *HindIII* fragments with the *Caprin1* TSS for cells isolated from anaemic spleen and kidney.

### CTCF and Rad21 are bound within regions of chromatin-chromatin interaction

**Figure 4:**
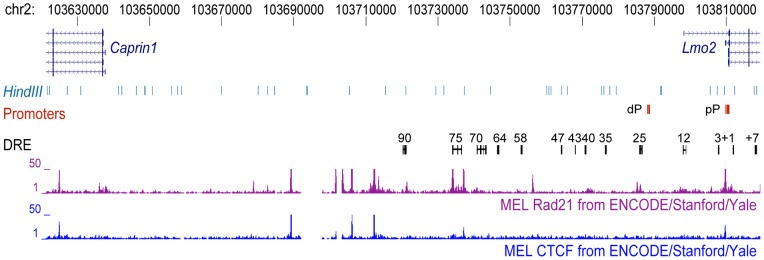
CTCF and RAD21 are bound within the *Lmo2-Caprin1* region at sites of chromatin-chromatin interaction. The mouse *Lmo2-Caprin1* region on chromosome 2 is depicted with chromosome coordinates shown at the top. *Lmo2* promoters are indicated by red boxes. Distal regulatory element (DRE) homology regions are indicated by black boxes joined by a line to delineate the human enhancer construct used in the generation of transgenic mice. Mouse ENCODE ChIP-Seq data for the cohesin complex member RAD21 and CTCF are shown below DRE. Proximal promoter (pP), distal promoter (dP), murine erythroleukemia cells (MEL differentiated with 2% DMSO).

Binding of CTCF and recruitment of the cohesin complex appears to be involved in many instances of chromatin-chromatin interactions at other loci [42], [43], [44], [45], [46], [47], [48]. Investigating ChIP-Seq data released by the mouse ENCODE project (Table S3) [61] we identified several CTCF and cohesin (RAD21) bound regions within the dCR (Figure 4) specifically within the fragment upstream of the 90 DRE. However, unlike the chromatin looping interactions, which we identified as being specific to erythroid cells, the CTCF bound region upstream of the 90 DRE was bound by CTCF in several cell types (Figure S4). Additional CTCF and cohesin bound regions are located just down-stream of the 75 DRE and at the *Lmo2* proximal promoter. Like the CTCF bound region upstream of the 90 DRE, CTCF was bound at the *Lmo2* proximal promoter in several cell types. CTCF bound 400 bp downstream of the 75 DRE was most prominent in erythroid cells but also observed in bone marrow, heart and lung tissues (Figure S4).

### Intergenic transcription at the distal regulatory elements

**Figure 5:**
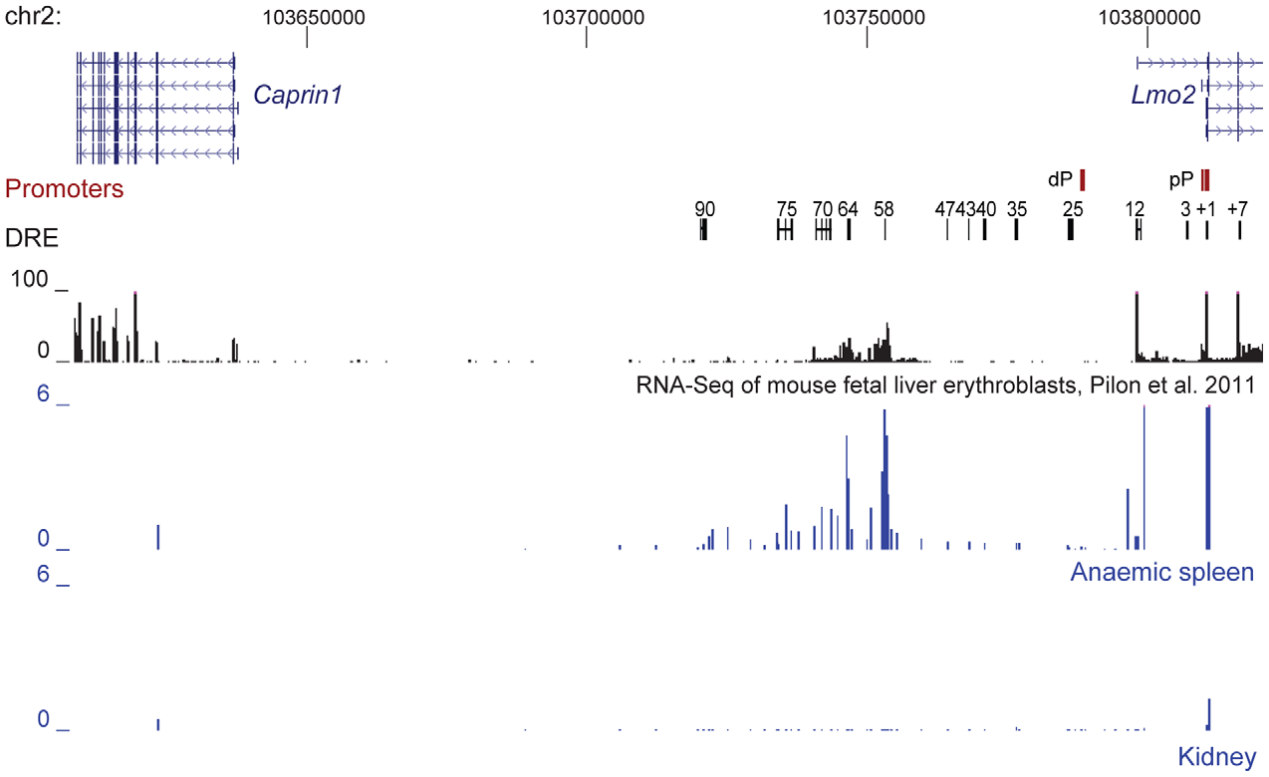
Intergenic transcription occurs upstream of *Lmo2* in erythroid cells. The mouse *Lmo2-Caprin1* region on chromosome 2 is depicted with chromosome coordinates shown at the top. RNA-Seq data for mouse fetal liver erythroblasts from Pilon et al. 2011 was obtained from the PSU Genome Browser (replicate 1 is shown in black). Transcript levels in adult mouse anaemic spleen and kidney were quantitatively assessed by RT-qPCR (shown in blue) and depicted relative to *Gapdh*. The levels downstream of 12 DRE (21.57) and the *Lmo2* pP (15.07) relative to *Gapdh* are off scale. Distal regulatory elements (DRE), distal promoter (dP), proximal promoter (pP).

Previous studies have identified intergenic transcripts throughout the human Hbb LCR which functions via a chromatin looping mechanism to regulate the β-globin genes [65], [66], [67]. In addition enhancer RNA (eRNA) has been identified at several neuronal enhancers [68]. Analysis of RNA-Seq data from mouse fetal liver erythroblasts [69] revealed intergenic transcription upstream of *Lmo2*, between the 58 and 70 DREs (Figure 5). To investigate intergenic transcription within this region in adult anaemic spleen erythroid cells and in kidney tissue we performed RT-qPCR. This analysis identified measurable levels of intergenic transcription occurring at all identified DRE in erythroid cells (Figure 5, Table S4). By contrast little or no intergenic transcription was detected in kidney tissue. In erythroid cells we detected the highest levels of intergenic transcription at the 58 DRE which diminished towards the 90 DRE, covering most of the dCR, whereas the intervening regions between the 58 and 12 DRE showed considerably lower levels of intergenic transcription.

## Discussion

Our investigation of chromatin-chromatin interactions throughout the mouse genomic region containing *Lmo2* and *Caprin1* identified several erythroid cell specific interactions between the *Lmo2* proximal promoter and upstream DRE. Specifically we identified chromatin-chromatin interactions in erythroid cells between the *Lmo2* proximal promoter and both a distal interaction cluster (dCR), containing three transcription factor bound DRE (70, 75, 90), and a proximal interaction cluster (pCR). Furthermore, 3C experiments revealed no significant interactions between the *Caprin1* TSS and the DRE suggesting these elements are specific in regulating *Lmo2* transcription.

Using a combination of ChIP-Seq data for mature erythroid cells and HPC7 hematopoietic progenitor cells we identified multiple transcription factors bound within both the pCR and the dCR. Even considering the HPC7 ChIP-Seq data that showed more transcription factor peaks across the entire region upstream of *Lmo2* the 75 DRE contained the highest density of transcription factor association as well as p300, RAD21 and a nearby CTCF bound region. Furthermore our 3C data confirmed specific interaction of the 75 DRE with the *Lmo2* proximal promoter and interaction of the *Lmo2* proximal promoter with the dCR containing the 75 DRE as well as the 90 and 70 DRE. In addition, a CTCF bound region upstream of the 90 DRE is contained in the dCR. Previous studies in circulating erythroid cells of transgenic mice show that the 75 DRE has the strongest enhancer activity in erythroid cells and drives the expression of a reporter gene, cooperatively with the *Lmo2* proximal promoter and +1 enhancer element [54]. We have shown that the 75 DRE functions via contacting the *Lmo2* proximal promoter/+1 DRE region to form an erythroid cell specific chromatin loop that includes other regulatory elements and a cluster of CTCF bound regions. This suggests that in the endogenous context elements throughout the dCR coordinately regulate *Lmo2* transcription in erythroid cells.

We also identified increased interaction throughout a pCR containing the 25 DRE/distal promoter and 12 DRE/intergenic promoter and the +1DRE/proximal promoter in erythroid cells compared to kidney cells, suggesting that this more proximal region also contributes to transcriptional regulation of endogenous *Lmo2*. Interestingly, we did not identify interactions of the 75 DRE with the fragments containing the 25 or 12 DRE. Transgenic analysis by Landry et al. 2009 of these elements did reveal that the 25 and 12 DRE conferred expression in the fetal liver whereas only the 75 DRE conferred expression in fetal liver as well as in circulating blood cells suggesting that these elements have different functional roles in regulating *Lmo2* expression. The fact that we did not identify specific interactions between the 75 DRE and the fragment containing the 25 or 12 DRE suggests that the interactions of these two regions with the proximal promoter are mutually exclusive. These mutually exclusive interactions could occur in different sub-populations of cells within the anaemic spleen or the interactions could be dynamic within individual cells with the proximal promoter region alternately contacting the 25–12 region and the 70–90 region similar to the flip-flop of the *Hbb* LCR between the γ- and β-genes [70].

We identified erythroid-cell specific chromatin loops between a dCR and the *Lmo2* proximal promoter; however the question remains as to which factors are mediating these looping interactions. LMO2 itself is one of the regulatory transcription factors bound to the upstream DRE, specifically at 75, 25 and 12, all of which showed increased interaction with the *Lmo2* promoter suggesting an important role for the LMO2 complex in chromatin looping. In support of this, a recent study found LDB1 (a member of the LMO2 complex) at regions of chromatin interaction with the LDB1 bound *Hbb-b1* promoter [57]. GATA1 and KLF1 are also bound within the distal interacting region, these transcription factors have been shown to be required, though not sufficient for chromatin looping between *Hbb-b1* and the LCR [44], [71], [72] and may have a similar role in regulating looping within the *Lmo2* locus. Cohesin (RAD21) is bound within both the dCR and pCR, specifically at the 75, 25 DRE as well as at the proximal promoter suggesting cohesin bound at the upstream DRE supports erythroid cell specific looping interactions with the *Lmo2* proximal promoter.

Cohesin is also recruited to CTCF occupied regions [46] and we identified several CTCF/RAD21 bound regions throughout the *Lmo2* upstream region, all of which occur within erythroid cell specific interacting domains, though the majority of the CTCF sites were not specific to erythroid cells. This is similar to the findings at the *Hbb* locus where CTCF bound regions, invariant between cell types, formed cell type specific chromatin loops [49]. CTCF is often associated with insulator activity; the CTCF/RAD21 occupied region upstream of the 90 DRE could have an important role in preventing *Caprin1* from interacting with the DRE that enhance *Lmo2* transcription in erythroid cells. As overexpression of *Caprin1* causes inhibition of cell division it is critical to prevent its aberrant upregulation in rapidly cycling erythroid cells [73]. In support of an important role for the CTCF bound region upstream of the 90 DRE this region is conserved and bound by CTCF in the human genome (not shown). We did identify one CTCF/RAD21 bound region, downstream of the 75 DRE, which may be critical in generating the tissue specific looping pattern that we identified. In summary, our results are consistent with chromatin-chromatin interactions throughout the *Lmo2* locus being supported by cohesin recruited both to CTCF bound regions (upstream of 90, downstream of 75) as well as at transcription factor and p300 bound enhancers not associated with CTCF (90, 70, 25, and 12 DRE).

Our analysis indicates that a large portion of the dCR is transcribed at moderate levels in erythroid cells but not in kidney. Whereas short eRNAs have been identified at enhancers in neuronal cells the broad domain of intergenic transcription we observed throughout the *Lmo2* dCR is more reminiscent of the human *Hbb* LCR [65], [66], [67], [68]. Intergenic transcription throughout the *Lmo2* dCR may function to facilitate the physical interaction of the dCR with the *Lmo2* proximal promoter through recruitment of both regions to a shared transcription factory. This co-localization at a shared transcription compartment would then allow transcription factors recruited to the dCR to influence the basal transcriptional machinery recruited to the *Lmo2* proximal promoter thereby enhancing *Lmo2* transcription.

In conclusion, we found that the mouse *Lmo2-Caprin1* locus adopts a tissue-specific conformation in erythroid cells. This tissue specific organization of the locus brings several DRE into proximity with the *Lmo2* proximal promoter while excluding the *Caprin1* TSS. A proximal control region, immediately upstream of the *Lmo2* proximal promoter, contains the 12 and 25 DRE as well as the distal and intermediate promoters and is more closely associated with the *Lmo2* proximal promoter in erythroid cells compared to kidney. Furthermore, a distal control region, covering 39 kb and containing three DRE (90, 75 and 70), forms a strong interaction with the *Lmo2* proximal promoter and shares many features with the well characterized *Hbb* LCR. A CTCF bound region upstream of the 90 DRE flanks the dCR and may function as an insulator preventing the interaction of *Caprin1* with the erythroid cell specific *Lmo2* enhancers.

## Materials and Methods

### Ethics Statement

All experiments were approved by the University Animal Care Committee (UACC) at the University of Toronto and the Bioscience Local Animal Care Committee (LACC).

### Cell Isolation

Adult globin expressing mature erythroid cells were isolated from C57/Blk6 mice in large numbers (>1×10^8^) from the spleen of mice 5 days after phenylhydrazine treatment. This treatment induces haemolytic anaemia, as a result of which the spleen becomes the major site of red blood cell production [62]. We disrupted fresh spleen or kidney tissue into a single-cell suspension and processed cells immediately as detailed below.

### Chromosome Conformation Capture (3C)

3C experiments were performed as described by Dekker et al. 2002 with the following modifications [74]. Formaldehyde was added to 2%, and the samples were cross linked for 10 minutes at room temperature. An overnight ligation of the digested DNA with T4 DNA ligase was performed. The 3C control template was prepared by mixing equimolar amounts of the BAC clone of the entire *Lmo2-Caprin1* locus (RP23-76D2) with the *Alpha Aortic Actin* 2 BAC clone (RP23-2N15) followed by digestion with *HindIII*. The digested DNA was then ligated, and purified using phenol/chloroform extraction and ethanol precipitation. *HindIII* restriction enzyme digestion efficiency was confirmed to be between 85 and 95% efficient at several genomic fragments in anaemic spleen and kidney cells (Figure S5).

The linear range of amplification was determined for erythroid and kidney samples by serial dilution. An appropriate amount of the DNA within the linear range (typically 40 ng of DNA) was subsequently used for quantification. PCR products of the ligated fragments were quantified using real-time quantitative PCR (qPCR) on the Bio-Rad CFX-384 cycler. All data points were generated from an average of three-five independent 3C experiments with the qPCR performed in triplicate. Standard curves were generated by 5 fold serial dilution of the 3C control template and run in parallel with 3C experimental samples. The primers used in qPCR are listed in Table S5. In each individual experiment 3C data were normalized to neighbouring fragments at the Alpha aortic actin locus (α-A2).

### RNA isolation and real-time RT-qPCR

RNA from anaemic spleen and kidney was isolated using TRIzol, according to the manufacturer`s instructions (Invitrogen). The iScript First strand synthesis cDNA kit from Bio-Rad was used for preparation of random-hexamer primed cDNA. Amplification in qPCR was performed on the Bio-Rad CFX-384 cycler using the standard curve method. The reaction mixture contained 2X Bio-Rad iTaq SYBR green mastermix, 0.3 pM of each primer, 1uL cDNA (10 times diluted from a 20 uL of reverse transcription reaction). The conditions for qPCR were as follows: 94°C for 3 min followed by 40 cycles at 94°C for 30 s, 62°C for 30 s. Expression levels of *Gapdh* or *Epn1* were used for normalization of expression levels. The primers used for real-time RT-qPCR are listed in Table S5.

### Statistical analysis

The 3C data were analyzed by two-way ANOVA using Sigma Plot12. Post tests (Holm-Sidak method) were performed to assess significant differences between anaemic spleen and kidney samples at specific genomic locations.

### Genome Mapping and Peak Identification from ChIP-Seq datasets in erythroid cells

ChIP-Seq raw data for GATA1, KLF1, LDB1, TAL1, and MTGR1 [57], [58], [59] listed in Table S3 were downloaded from Gene Expression Omnibus (GEO) [75]. ChIP-Seq data were aligned to NCBIm37 mouse assembly (mm9) using Bowtie alignment [76] by suppressing alignments to only 1 best reportable alignment with a maximum number of 2 mismatches within 28 nucleotides of seed length in the high quality end. The SISSRs [77] algorithm was subsequently used to identify significant transcription factor peaks compared to that of the input DNA with p <0.001. To remove amplification bias, multiple reads aligning to the same genomic coordinate were counted as one. Parameters for the corresponding transcription factor data were set according to original publications using applicable input data sets. Significant transcription factor peaks were uploaded to UCSC genome browser for visualization [78]. The HPC7 ChIP-Seq data analysis was performed by using published peaks [64]. ChIP-Seq data for CTCF, p300, RAD 21, H3K4me1, DNaseI hypersensitivity data were obtained from the mouse ENCODE project (Table S3) [61].

## Supporting Information

Figure S1 **The** ***Lmo2*** **/** ***Caprin1*** **region on mouse chromosome 2.** Primers used in chromosome conformation capture (3C) and *HindIII* restriction sites are shown across the *Lmo2*/*Caprin1* region of mouse chromosome 2. Promoters and distal regulatory elements (DRE) are depicted in red and black respectively. Anchor fragments used in the *Caprin1*, 75 DRE and *Lmo2* 3C experiments are marked with an asterisk (*). Distal promoter (pP), proximal promoter (pP).
(PDF)

Figure S2 **Distal regulatory elements upstream of** ***Lmo2*** **overlap transcription factor bound regions in HPC7 hematopoietic progenitor cells.** The mouse *Lmo2-Caprin1* region. Distal regulatory element (DRE) homology regions are indicated by black boxes joined by a line to delineate the human enhancer construct used in the generation of transgenic mice. Coloured boxes represent peaks identified from transcription factor ChIP-Seq data from HPC7 hematopoietic progenitor cells obtained from Wilson et al. 2010. Proximal promoter (pP), distal promoter (dP).
(PDF)

Figure S3 **The** ***Caprin1*** **TSS does not interact with distal regulatory elements upstream of** ***Lmo2*** **.** Quantitative chromosome conformation capture (3C) was performed to detect chromatin-chromatin interactions between the *Caprin1* TSS and distal regulatory elements (DRE). The profile of interactions identified in anaemic spleen (red) and kidney (blue) is displayed. Black box indicates the anchor fragment at *Caprin1* and alternating intensities of grey boxes indicate the fragments investigated for interactions. Data points are an average of three independent biological replicates. Error bars depict the SEM, no significant differences were identified throughout this region.
(PDF)

Figure S4 **CTCF bound upstream of** ***Lmo2*** **in different cell types.** The mouse *Lmo2* upstream region on chromosome 2 is depicted with chromosome coordinates shown at the top. *HindIII* restriction sites are indicated by blue lines. The two *Lmo2* promoters are indicated by red boxes. Distal regulatory element (DRE) homology regions are indicated by black boxes joined by a line to delineate the human enhancer construct used in the generation of transgenic mice. Mouse ENCODE ChIP-Seq data from B Ren (Ludwig Inst. for Cancer Research) and M Snyder (Stanford University) for CTCF in different cell types are shown below the DRE. Proximal promoter (pP), distal promoter (dP), murine erythroleukemia cells (MEL differentiated with 2% DMSO), bone marrow (BM), embryonic stem cells (ES-Bruce4), mouse embryonic fibroblasts (MEF).
(PDF)

Figure S5 **Restriction digestion efficiency in chromosome conformation capture.** Restriction digestion efficiency was between 85 and 95% at several *HindIII* restriction sites. *Lmo2* proximal promoter (pP), Distal regulatory element (DRE), *Caprin1* promoter (Caprin1P). “U” denotes a restriction fragment upstream of the indicated element.
(PDF)

Table S1 **Coordinates of distal regulatory elements located upstream of the** ***Lmo2*** **promoter in the mouse genome.** Distal regulatory elements are named acording to their distance upstream of the annotated *Lmo2* transcription start site overlapping the proximal promoter. Coordinates are given for homology regions identified by BLAT. All fragments were mapped in NCBI m37 mouse assembly (mm9).
(PDF)

Table S2 **Coordinates of the** ***Lmo2*** **proximal and distal promoters in the mouse genome.** The coordintes of proximal promoters and distal promoters for the *Lmo2* gene in the mouse genome are listed in the table. Coordinates are given for homology regions identified by BLAT. All fragments were mapped in NCBI m37 mouse assembly (mm9).
(PDF)

Table S3 **Chromatin immunoprecipitation sequencing data.** Transcription factor binding sites have been obtained from three different cell types; differentiated murine erythroleukemia cells (MEL), hematopoietic progenitor cells (HPC7), and GIE-ER4 a GATA1-null erythroblast cell line in which GATA1 activity was restored. CTCF, DNaseI hypersensitivity, p300 and RAD21 data have been obtained from the mouse ENCODE project, sources listed (Principal investigator, Institution).
(PDF)

Table S4 **Relative transcript abundance in intergenic regions upstream of** ***Lmo2*** **.**
(PDF)

Table S5 **Primers.** Specific primers are listed for the chromosome conformation capture (3C) and RT-qPCR analyses. Left primer (L), right primer (R), primers used to test *HindIII* restriction digestion efficiency are marked as REX.
(PDF)
