# Supplementary figures and images for: Upstream Distal Regulatory Elements Contact the Lmo2 Promoter in Mouse Erythroid Cells
Source: PLoS One. 2012 Dec 21;7(12):e52880. doi: 10.1371/journal.pone.0052880 (PMC3528669; doi:10.1371/journal.pone.0052880)

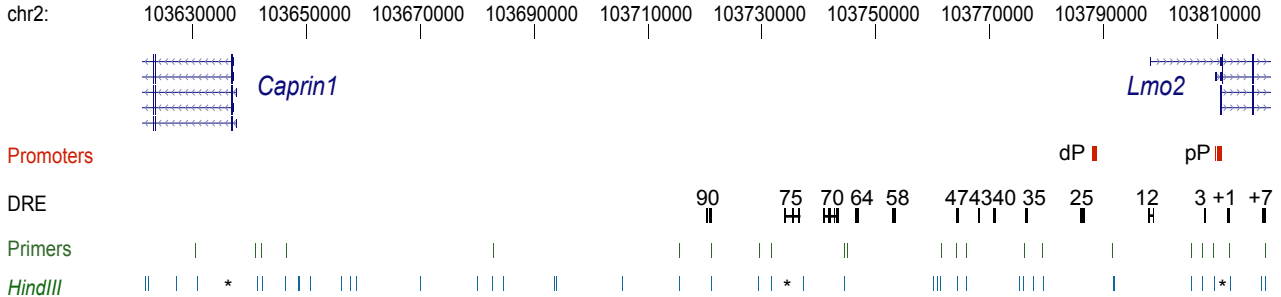

Supplement: Figure S1 — The Lmo2 / Caprin1 region on mouse chromosome 2. Primers used in chromosome conformation capture (3C) and HindIII restriction sites are shown across the Lmo2/Caprin1 region of mouse chromosome 2. Promoters and distal regulatory elements (DRE) are depicted in red and black respectively. Anchor fragments used in the Caprin1, 75 DRE and Lmo2 3C experiments are marked with an asterisk (*). Distal promoter (pP), proximal promoter (pP). (PDF) [file pone.0052880.s001.pdf]

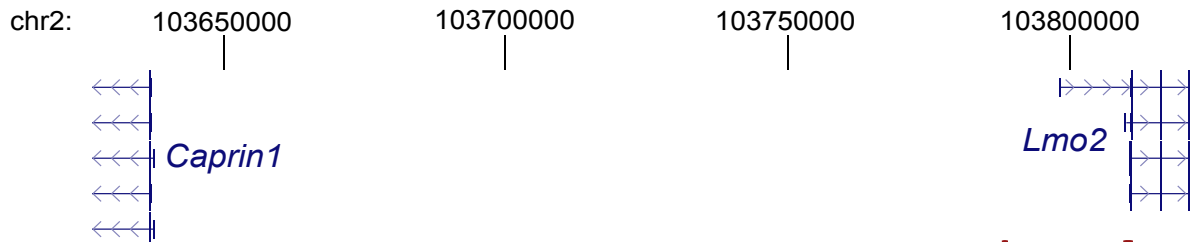

Promoters

dP pP

DREs

90 75 70 64 58 47 43 40 35 25 12 3+1 +7

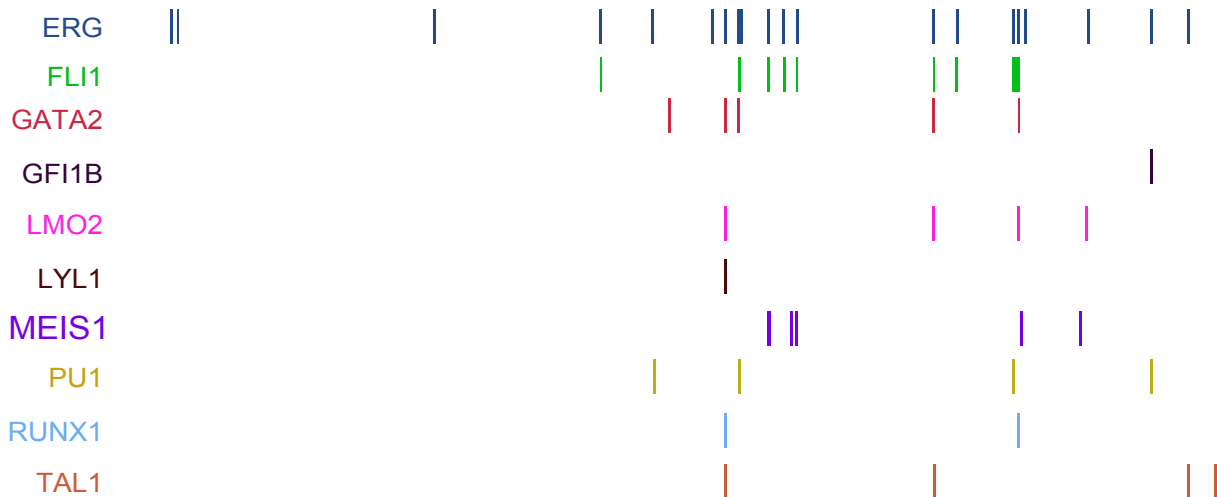

Supplement: Figure S2 — Distal regulatory elements upstream of Lmo2 overlap transcription factor bound regions in HPC7 hematopoietic progenitor cells. The mouse Lmo2-Caprin1 region. Distal regulatory element (DRE) homology regions are indicated by black boxes joined by a line to delineate the human enhancer construct used in the generation of transgenic mice. Coloured boxes represent peaks identified from transcription factor ChIP-Seq data from HPC7 hematopoietic progenitor cells obtained from Wilson et al. 2010. Proximal promoter (pP), distal promoter (dP). (PDF) [file pone.0052880.s002.pdf]

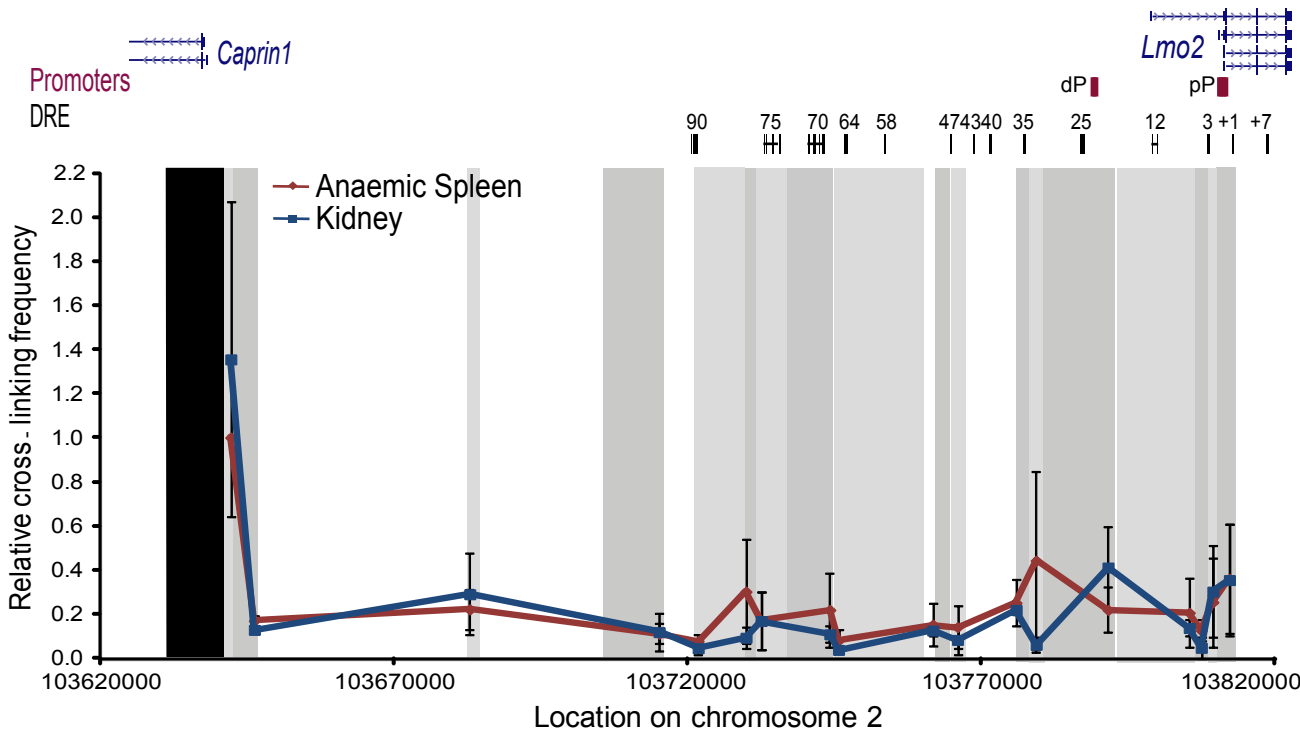

Supplement: Figure S3 — The Caprin1 TSS does not interact with distal regulatory elements upstream of Lmo2 . Quantitative chromosome conformation capture (3C) was performed to detect chromatin-chromatin interactions between the Caprin1 TSS and distal regulatory elements (DRE). The profile of interactions identified in anaemic spleen (red) and kidney (blue) is displayed. Black box indicates the anchor fragment at Caprin1 and alternating intensities of grey boxes indicate the fragments investigated for interactions. Data points are an average of three independent biological replicates. Error bars depict the SEM, no significant differences were identified throughout this region. (PDF) [file pone.0052880.s003.pdf]

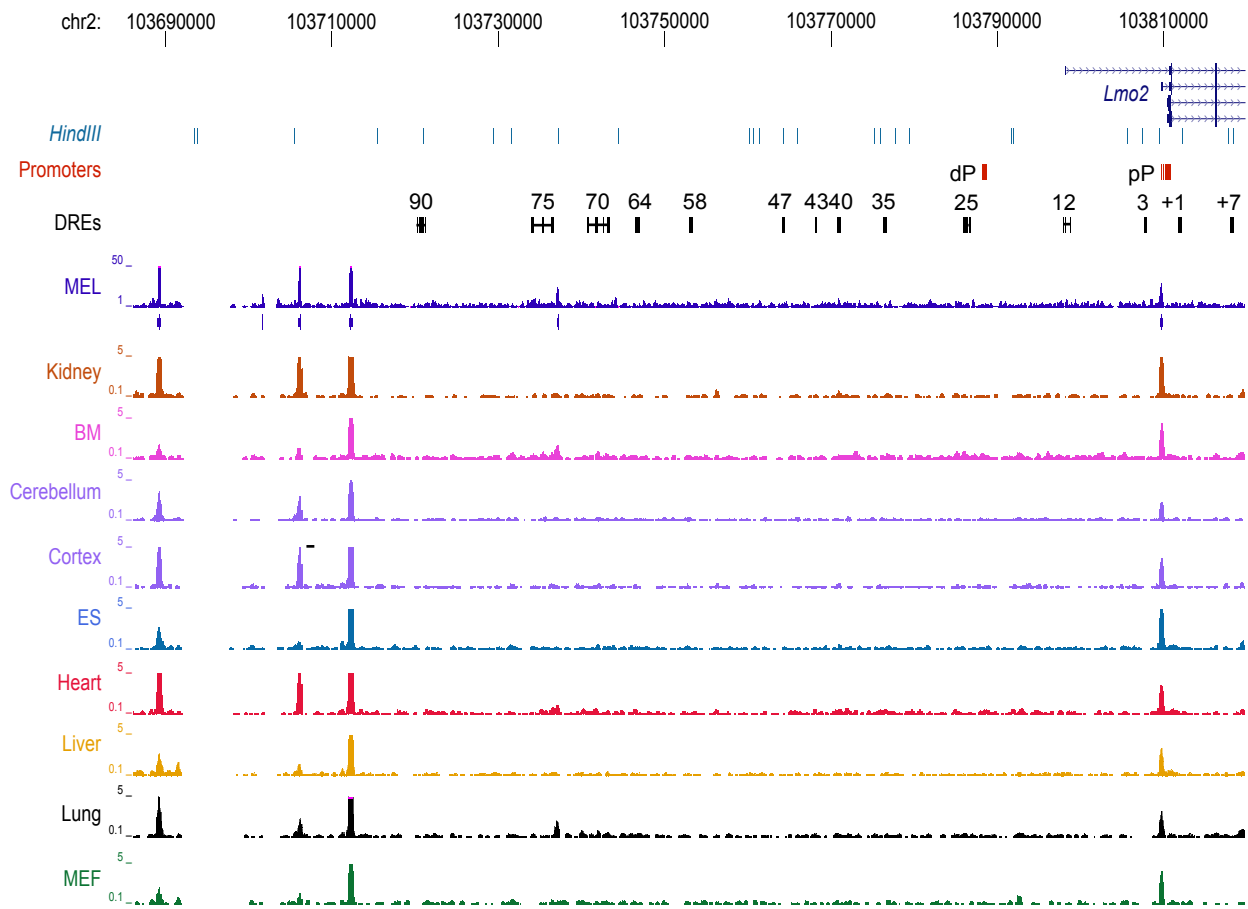

Supplement: Figure S4 — CTCF bound upstream of Lmo2 in different cell types. The mouse Lmo2 upstream region on chromosome 2 is depicted with chromosome coordinates shown at the top. HindIII restriction sites are indicated by blue lines. The two Lmo2 promoters are indicated by red boxes. Distal regulatory element (DRE) homology regions are indicated by black boxes joined by a line to delineate the human enhancer construct used in the generation of transgenic mice. Mouse ENCODE ChIP-Seq data from B Ren (Ludwig Inst. for Cancer Research) and M Snyder (Stanford University) for CTCF in different cell types are shown below the DRE. Proximal promoter (pP), distal promoter (dP), murine erythroleukemia cells (MEL differentiated with 2% DMSO), bone marrow (BM), embryonic stem cells (ES-Bruce4), mouse embryonic fibroblasts (MEF). (PDF) [file pone.0052880.s004.pdf]

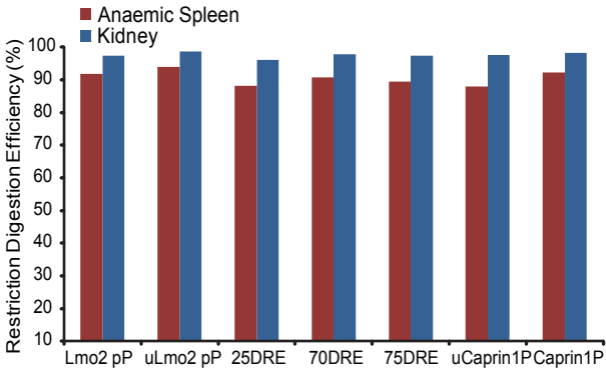

Supplement: Figure S5 — Restriction digestion efficiency in chromosome conformation capture. Restriction digestion efficiency was between 85 and 95% at several HindIII restriction sites. Lmo2 proximal promoter (pP), Distal regulatory element (DRE), Caprin1 promoter (Caprin1P). “U” denotes a restriction fragment upstream of the indicated element. (PDF) [file pone.0052880.s005.pdf]
