# Supplementary material for: Upstream Distal Regulatory Elements Contact the Lmo2 Promoter in Mouse Erythroid Cells
Source: PLoS One. 2012 Dec 21;7(12):e52880. doi: 10.1371/journal.pone.0052880 (PMC3528669; doi:10.1371/journal.pone.0052880)
Supplement: Table S1 — Coordinates of distal regulatory elements located upstream of the Lmo2 promoter in the mouse genome. Distal regulatory elements are named acording to their distance upstream of the annotated Lmo2 transcription start site overlapping the proximal promoter. Coordinates are given for homology regions identified by BLAT. All fragments were mapped in NCBI m37 mouse assembly (mm9). (PDF) [file pone.0052880.s006.pdf]

Distal Regulatory Element	Chromosome	Starts at	Ends at
**90**	**Chr2**	103720746	103720876
	**Chr2**	103720495	103720607
	**Chr2**	103720233	103720316
	**Chr2**	103720614	103720670
	**Chr2**	103720684	103720729
	**Chr2**	103720909	103720959
**75**	**Chr2**	103736385	103736662
	**Chr2**	103733907	103733995
	**Chr2**	103734073	103734138
	**Chr2**	103741698	103741839
	**Chr2**	103740691	103740810
**70**	**Chr2**	103743108	103743214
	**Chr2**	103743323	103743439
	**Chr2**	103740817	103740859
	**Chr2**	103743246	103743284
	**Chr2**	103742632	103742668
	**Chr2**	103743288	103743312
	**Chr2**	103741882	103741906
	**Chr2**	103741847	103741854
**64**	**Chr2**	103746643	103746795
	**Chr2**	103746829	103746975
	**Chr2**	103746393	103746425
	**Chr2**	103746616	103746634
	**Chr2**	103746616	103746634
**58**	**Chr2**	103753051	103753386
	**Chr2**	103753006	103753038
**47**	**Chr2**	103764161	103764463
**43**	**Chr2**	103768003	103768252
**40**	**Chr2**	103770972	103771182
	**Chr2**	103770692	103770883
	**Chr2**	103770897	103770953

**35**	**Chr2**	103776285	103776669
	**Chr2**	103776677	103776702
**25**	**Chr2**	103786638	103786800
	**Chr2**	103786084	103786206
	**Chr2**	103785920	103785949
	**Chr2**	103786575	103786598
	**Chr2**	103786541	103786557
	**Chr2**	103786363	103786383
	**Chr2**	103785810	103785823
	**Chr2**	103786233	103786243
	**Chr2**	103786568	103786573
**12**	**Chr2**	103798740	103798811
	**Chr2**	103797914	103797994
	**Chr2**	103798059	103798101
**3**	**Chr2**	103807666	103807799
	**Chr2**	103807840	103807879
	**Chr2**	103807626	103807654
	**Chr2**	103807906	103807921
**+1**	**Chr2**	103811961	103812138
	**Chr2**	103811728	103811830
	**Chr2**	103811901	103811939
	**Chr2**	103811837	103811880
**+7**	**Chr2**	103818142	103818313
	**Chr2**	103818037	103818138
	**Chr2**	103817938	103817999
	**Chr2**	103818354	103818374
	**Chr2**	103818324	103818338
	**Chr2**	103818018	103818031
