# Supplementary material for: Upstream Distal Regulatory Elements Contact the Lmo2 Promoter in Mouse Erythroid Cells
Source: PLoS One. 2012 Dec 21;7(12):e52880. doi: 10.1371/journal.pone.0052880 (PMC3528669; doi:10.1371/journal.pone.0052880)
Supplement: Table S2 — Coordinates of the Lmo2 proximal and distal promoters in the mouse genome. The coordintes of proximal promoters and distal promoters for the Lmo2 gene in the mouse genome are listed in the table. Coordinates are given for homology regions identified by BLAT. All fragments were mapped in NCBI m37 mouse assembly (mm9). (PDF) [file pone.0052880.s007.pdf]

Promoter Element	Chromosome	Starts at	Ends at
Distal Promoter (dp)	**Chr2**	103788235	103788347
	**Chr2**	103788107	103788162
	**Chr2**	103788392	103788432
	**Chr2**	103788641	103788683
	**Chr2**	103788499	103788515
	**Chr2**	103788081	103788091
	**Chr2**	103788372	103788379
	**Chr2**	103788202	103788208
	**Chr2**	103788222	103788228
	**Chr2**	103788168	103788172
Proximal promoter (pP)	**Chr2**	103810212	103810400
	**Chr2**	103810495	103810533
	**Chr2**	103810398	103810445
Proximal promoter extended (pPex)	**Chr2**	103810212	103810400
	**Chr2**	103810620	103810724
	**Chr2**	103810734	103810812
	**Chr2**	103809864	103809907
	**Chr2**	103810495	103810545
	**Chr2**	103809630	103809688
	**Chr2**	103810398	103810445
	**Chr2**	103810140	103810170
