# Supplementary material for: Upstream Distal Regulatory Elements Contact the Lmo2 Promoter in Mouse Erythroid Cells
Source: PLoS One. 2012 Dec 21;7(12):e52880. doi: 10.1371/journal.pone.0052880 (PMC3528669; doi:10.1371/journal.pone.0052880)
Supplement: Table S3 — Chromatin immunoprecipitation sequencing data. Transcription factor binding sites have been obtained from three different cell types; differentiated murine erythroleukemia cells (MEL), hematopoietic progenitor cells (HPC7), and GIE-ER4 a GATA1-null erythroblast cell line in which GATA1 activity was restored. CTCF, DNaseI hypersensitivity, p300 and RAD21 data have been obtained from the mouse ENCODE project, sources listed (Principal investigator, Institution). (PDF) [file pone.0052880.s008.pdf]

Protein or Chromatin Feature	Cell type	References
LDB1	Differentiated MEL	(Soler et al., 2010)
TAL1		
MTGR1		
GATA1		
GATA1	GIE-ER4 cells	(Cheng et al., 2009)
H3K4me1	GIE-ER4 cells	ENCODE (R Hardison, Penn State University)
KLF1	e14.5 fetal livers	(Tallack et al. 2010)
ERG	HPC7 Hematopoietic Progenitor Cells	(Wilson et al., 2010)
FLI1		
RUNX1		
LYL1		
MEIS1		
PU1		
GATA2		
GFI1b		
TAL1		
LMO2		
p300	MEL	ENCODE (M Snyder, Stanford University)
CTCF	MEL (2% DMSO)	
RAD21		
DNaseI hypersensitivity	MEL, Kidney	ENCODE (JA Stamatoyannopoulos, University of Washington)
CTCF	Various	ENCODE (B Ren, Ludwig Inst. for Cancer Research)
