# Supplementary material for: Upstream Distal Regulatory Elements Contact the Lmo2 Promoter in Mouse Erythroid Cells
Source: PLoS One. 2012 Dec 21;7(12):e52880. doi: 10.1371/journal.pone.0052880 (PMC3528669; doi:10.1371/journal.pone.0052880)
Supplement: Table S4 — Relative transcript abundance in intergenic regions upstream of Lmo2 . (PDF) [file pone.0052880.s009.pdf]

Chromosome	Starts at	Ends at	Erythroid	Kidney
chr2	103719773	103719855	0.07	0.00
chr2	103720768	103720926	0.19	0.00
chr2	103721765	103721936	0.52	0.00
chr2	103733981	103734083	0.00	0.00
chr2	103734036	103734162	0.20	0.00
chr2	103736359	103736503	0.76	0.00
chr2	103733874	103733987	0.70	0.02
chr2	103735453	103735544	1.83	0.00
chr2	103737677	103737813	0.72	0.00
chr2	103741899	103741916	1.74	0.00
chr2	103743566	103743701	1.67	0.02
chr2	103740379	103740594	0.98	0.01
chr2	103744683	103744817	1.40	0.02
chr2	103747146	103747258	0.83	0.00
chr2	103753011	103753170	5.77	0.04
chr2	103753441	103753558	4.69	0.03
chr2	103764283	103764406	0.32	0.03
chr2	103768070	103768257	0.32	0.00
chr2	103770958	103771051	0.24	0.01
chr2	103776571	103776653	0.25	0.14
chr2	103776976	103777175	0.25	0.04
chr2	103785900	103786097	0.09	0.01
chr2	103787110	103787196	0.03	0.00
chr2	103785656	103785825	0.16	0.00
chr2	103796380	103796571	2.50	0.02
chr2	103797876	103798028	0.55	0.00

chr2	103799435	103799515	21.57	0.07
chr2	103788221	103788401	0.14	0.02
chr2	103788891	103788984	0.06	0.01
chr2	103810515	103810638	5.89	0.20
chr2	103746604	103746751	2.95	0.01
chr2	103798226	103798325	0.55	0.00
chr2	103712293	103712439	0.15	0.00
chr2	103705781	103705877	0.15	0.00
chr2	103688916	103689025	0.04	0.01
chr2	103810841	103811055	15.07	1.28
chr2	103623318	103623538	1.00	0.43
chr2	103746245	103746326	4.73	0.03
chr2	103722341	103722435	0.84	0.00
chr2	103725075	103725176	0.9	0.00
chr2	103731605	103731706	0.18	0.00
chr2	103752577	103752695	3.2	0.00
chr2	103792261	103792371	0.00	0.00
chr2	103794182	103794283	0.00	0.00
chr2	103729128	103729266	0.41	0.00
chr2	103749894	103749988	0.42	0.00
chr2	103750643	103750736	1.71	0.03
chr2	103754295	103754405	0.83	0.00
chr2	103755261	103755406	0.69	0.00
chr2	103759636	103759735	0.43	0.03
chr2	103753751	103753850	2.26	0.00
