# Supplementary material for: Upstream Distal Regulatory Elements Contact the Lmo2 Promoter in Mouse Erythroid Cells
Source: PLoS One. 2012 Dec 21;7(12):e52880. doi: 10.1371/journal.pone.0052880 (PMC3528669; doi:10.1371/journal.pone.0052880)
Supplement: Table S5 — Primers. Specific primers are listed for the chromosome conformation capture (3C) and RT-qPCR analyses. Left primer (L), right primer (R), primers used to test HindIII restriction digestion efficiency are marked as REX. (PDF) [file pone.0052880.s010.pdf]

3C primers	Sequence 5`-3`
LMO2-3C-pP-L	AGGAGAGAAACAACAACCCTTT
LMO2-3C-upstream pP-L	GGGGACCTAGGTTTTTCTCCT
LMO2-3C-downstream 12E-L	TTCAGACTTCTGACATCCTTATTTC
LMO2-3C-12E-L	CTGCCTTACCTTGAGCTTGG
LMO2-3C-25E-L	ACCCTTGGCAATTAACGTGT
LMO2-3C-downstream 35E-L	TGTGGAACACCAACTTTTCCT
LMO2-3C-35E-L	CTGGGCCAAGGGGTATAGAG
LMO2-3C-47E-L	CTACTCCCGCTCAAAACTGC
LMO2-3C-downstream 58E-R	CTTGGTACCCAGGAACTAGCA
LMO2-3C-64E-R	CTCCCCTCCCTCAAACATTA
LMO2-3C-70E-L	GGACTACGGAGCTGAAACCA
LMO2-3C-75E-R	CCCTACAACATGCATCTCCA
LMO2-3C-upstream 75E-R	TGCTTGATCATGGTTACAGGTC
LMO2-3C-downstream 90E-R	TTGGGGTCATTATCTCTTTGCT
LMO2-3C-90E-L	GGCCCTTATAATTTGGCACA
LMO2-3C-blankregion1-L	TGGCACACATCTACAAGAGCA
LMO2-3C-blankregion2-L	TCTCTGAACTGTTCCCTGGAG
LMO2-3C-upstream Caprinpr-L	TTTCATCAAGTGCATCTTTGC
LMO2-3C-Caprinpr-L	CCAGAGAGGCTGTTGGTTACT
LMO2-3C-downstream Caprinpr-L	CATTGGTATGTTCATTACCTAGACA
Alpha aortic actinHIII-4-3C-L	CCCTAGTCAGCCATCTCCTCT
Alpha aortic actinHIII-5-3C-L	TGCAGTTATGTTCCACAGCAG
RT-qPCR Primers	Sequence 5`-3`
CTCF peak1-L	ATGCTGGTTTGTCATCTCCTGA
CTCF peak1-R	AGTGCATGAGGATGTGCAATTA

CTCF peak 2-L	ACTCACAGATTTGCTGGAGAGAC
CTCF peak 2-R	TGGTGATTAACCAACTTCAGACA
CTCF peak 3-L	GGTTGCTATGGTTGCAGATAGAG
CTCF peak3-R	CAGGAGTTGTGTAGACCGAGAAT
u90-L	TCATCAGCACTTACAGCCTCA
u90-R	ATATGGCTGCAACAATTTCTGA
90DRE-L	ATTCTTGTTTATGTAGGGGTGATGT
90DRE-R	GATACCATAAAATCAGAGGCAGGTA
d90-L	CCCCCTTTAGAGTACTGCACTG
d90-R	CCAGATACGATGCCTGTGATAG
d90A-L	TCTTCCAGTTCAATATGCTCCTAAC
d90A-R	GGAGAGGTCTGATACAGTCGTTTTA
d90BL	ATATTTAGAAAGGCCAGAATTTTGCT
d90B-R	GTTTGGGAATTATAGCCCTACGATA
90-75A-L	ACTGAGGAAGTGCAGCAGATTAAC
90-75A-R	AAGAATTTCAGCGAACTCTAAGGA
u75A-L	GCAGGTGGTATTGTTTAGTGAGGTA
u75A-R	AGAAGCATGGGGTAGTGGATT
u75B-L	ACCAAGCGGAGGCTGTATTA
u75B-R	TTAGCTGCCTCAGAAGATAATGG
75DRE-L	CAGCTAACTGTTACAGGAGAAGGAG
75DRE-R	TGGGATCTGGGAGAGTATACTACAG
d75-L	GGAAGTAAGGGAGACCCATTG
d75-R	TTTGCTAGAAATCCCAACGTG
u70-L	AACTATGGGGAGCATAAGCAAA
u70-R	CAGGCAAATATCTAGGGGAAAA

70DRE-L	AAAGGGGCCAGCTAGGAG
70DRE-R	CTCAACCTGTTGGCGTATCC
47DRE-R	GTCCGAACCTTTCAGTGTTCTC
d70-R	TACTGGGTTAAAGAAGGGGTGA
u64A-L	ACCAAGTTGGCTAAGGGTAGTTTT
u64B-L	GAGGAGCCAGAGTTAAACCAAGT
u64AB-R	GGACACCTAATAACGTGTTAGGATTAG
u64-L	GGCTAAGGGTAGTTTTGCAGAG
u64-R	CGATCAGACTGAGTGTGTGAGA
64DRE-L	AAGGATCAGTGTGGAACTTGC
64DRE-R2	TGGCGACAGCACAGAAATAG
d64-L	CCTGCTGTTTATGCAACACTTC
d64-R	GCCTAACAAACTGGGATTCACT
64-58ER	GGTAGCAATCTGGATATCTTGGAG
64-58EL	ATGTATCCTTCAGAGGAGGCATAG
64-58CR	TCTGCTTAATTGTTGGGCCTCT
64-58CL	TAAGTCAACCTGCCGTTAATTGTA
u58-L	AATAAGAGAGGAGAACGCAGTATGA
u58-R	CATGTTTAGAAACAGAGGGTTATGC
58DRE-L	TTCAGAACTCCCCGAAGAGA
58DRE-R	CTCAGTTCCAAACCGCTCAG
d58-L	TGGCATTGATTTTCCCTATTTT
d58-R	GCCCTGTACCTACCTCAAGATG
58-47AR	TCCTTTTACGGAACATGATGAACT
58-47AL	AGAGTGACTTCAATTTGGACCATT
58-47BR	CATTTTAGCTTCCCAAATGGTTAT

58-47BL	AGATACGTGACCTAAACAGCATTC
58-47CL	GAAGACTGCCTCGGTTTATTCTTA
58-47CR	ATTTACACCTTGTCCTGATTTCGT
58-47DL	GTCTATAACACAGATGACCCATGC
58-47DR	AGTTTGGGAACAATCAGAAGCTAT
47DRE-L	CAGTGCATGGAGTTAATGGAAA
47DRE-R	ACTACAACTTGGTGCTGGCAAT
43DRE-L	GTGGGCCAATTAGTGTCTGG
43DRE-R	CCCCAGGCTTTGTTCTACATT
40DRE-L	GAGGGAGGGAGTTCGTAACA
40DRE-R	AATAATGAATGCGCGTCTCC
35DRE-L	GGCATGATCGATACAAGACAGA
35DRE-R	GCACTTAAATGGAACTCCCAAC
d35-L	GCCACATACCATCTAAACAGCA
d35-R	CTACTGGTGCCCTGTCCTACTC
u25-L	ATGACTGGATTCACCACCTTG
u25-R	GCTAACCACATCAAACCAACC
25DRE-L	GGGGATGAATGCATGATAGACT
25DRE-R	GGCTGAAGGGAAACTGTGTAAC
d25-L	AGAACAGCCAGGTGAGATGAA
d25-R	AGGCATCATCCTAACCAGTGA
u12-L1	CTTTTCAACTCCCGGAGGAT
u12-R1	GGGAGAGGTACCTTCTTCAAGC
u12-L2	GAAGTACTGCGGTCCTTGATATG
u12-R2	TATTCTTATACAAGCATGGGCATC
12DRE-L	GCAAAAAGTTGCCAGATAAAAGATA

12DRE-R	ACATTGTAAGTCTTCGAGGTAGGTG
d12-L	GGGATGTTAAAAGGGATCCTG
d12-R	CATGAGCGAGCAGAATTTGAC
dP_Int_Lmo2-L	ACTTTGCTGACTTCCACAAGGAC
dP_Int_Lmo2-R	GATGTAATCCCTGTGACTCCTGAT
d-dPL1	CTAAAGTCACGAGAAGGACCAAA
d-dPR1	CCAAAGACTCCTTACTTGCTCAG
d-dPL2	CTGCACCCTAGATGAATAACACC
d-dPR2	ACTGTTTGGGTATGCTACACTCG
d-dPL3	AAGGACTTGGAATAACCTTGCTAGT
d-dPR3	TGGTAGTAGGAACACTCTCTCGTCT
pP_Int_Lmo2-L	GATGGAAGGTTAAGTCCTGAGCA
pP_Int_Lmo2-R	AAAGAGAGAGAGCGAATCATCCAG
Lmo2 Exon2-L2	ATCGAAAGGAAGAGCCTGGAC
Lmo2 Intron2 -R2	GGTCGATCCCAGTTACAGCTTC
Pkd2_In2-L	GGAGGGAAAGAGCTGACCTTA
Pkd2_EX3-R	AGCTCATCATGCCGTAGGTC
Vh16 genic-L	GGAGGGTCCACTAAACTCTCTTG
Vh16 genic-R	GCATAGCCTTTTCCACTCTCATC
GapdhE1I1-L	CTTCTTGTGCAGTGCCAGGTG A
GapdhE1I1-R	CGCACCAGCATCCCTAGACC
Slc4a1E1I1-L	TGGGAGCTCAGCCAGTCACA
Slc4a1E1I1-R	CGGGACAGATGCCAA AGGAC
Caprin1E3-L2	CCTTTCCCCTTTATTCATTCG
Caprin1I2-R2	AGCAATGGTCAGTGTTTCAAGTT
EpnE1I1-L	CTGGAAGCCCGGTATAAGC

EpnE1I1-R	GTACAAAAGCAGCCACAAGC
LMO2 pP REX -L	AGGAGAGAAACAACAACCCTTT
LMO2 pP REX -R	TGCCTCCCCAACTGTGTAAT
ULmo2 pPREX-L	GGGGACCTAGGTTTTTCTCCT
ULmo2 pPREX-R	GGAAGTTCCTTCCCGATAAAA
25E REX -R	TTTGGCTGATGCAGAGAATG
25E REX -L	ACCCTTGGCAATTAACGTGT
70E REX -L	GGACTACGGAGCTGAAACCA
70E REX -R	CTCCCCTCCCTCAAACATTA
75E REX -R	AGCCAGGCACAAATTACCTC
75E REX -L	GTGGCACTCTCTGCTGACC
UCaprin1 REX -R	TCCCTGTCAAACTGATGCAC
UCaprin1 REX -L	TTTCATCAAGTGCATCTTTGC
Caprin1pr REX -R	TTTCCCAAGTAGGTCCCTGA
Caprin1pr REX -L	CCAGAGAGGCTGTTGGTTACT
